# Microarray Analyses of Glucocorticoid and Vitamin D3 Target Genes in Differentiating Cultured Human Podocytes

**DOI:** 10.1371/journal.pone.0060213

**Published:** 2013-04-04

**Authors:** Xiwen Cheng, Xuan Zhao, Simran Khurana, Leslie A. Bruggeman, Hung-Ying Kao

**Affiliations:** 1 Department of Biochemistry, School of Medicine, Case Western Reserve University (CWRU) and the Comprehensive Cancer Center of CWRU, Cleveland, Ohio, United States of America; 2 Rammelkamp Center for Education and Research and Department of Medicine, MetroHealth Medical Center, Case Western Reserve University School of Medicine, Cleveland, Ohio, United States of America; University of Houston, United States of America

## Abstract

Glomerular podocytes are highly differentiated epithelial cells that are key components of the kidney filtration units. Podocyte damage or loss is the hallmark of nephritic diseases characterized by severe proteinuria. Recent studies implicate that hormones including glucocorticoids (ligand for glucocorticoid receptor) and vitamin D3 (ligand for vitamin D receptor) protect or promote repair of podocytes from injury. In order to elucidate the mechanisms underlying hormone-mediated podocyte-protecting activity from injury, we carried out microarray gene expression studies to identify the target genes and corresponding pathways in response to these hormones during podocyte differentiation. We used immortalized human cultured podocytes (HPCs) as a model system and carried out *in vitro* differentiation assays followed by dexamethasone (Dex) or vitamin D3 (VD3) treatment. Upon the induction of differentiation, multiple functional categories including cell cycle, organelle dynamics, mitochondrion, apoptosis and cytoskeleton organization were among the most significantly affected. Interestingly, while Dex and VD3 are capable of protecting podocytes from injury, they only share limited target genes and affected pathways. Compared to VD3 treatment, Dex had a broader and greater impact on gene expression profiles. In-depth analyses of Dex altered genes indicate that Dex crosstalks with a broad spectrum of signaling pathways, of which inflammatory responses, cell migration, angiogenesis, NF-κB and TGFβ pathways are predominantly altered. Together, our study provides new information and identifies several new avenues for future investigation of hormone signaling in podocytes.

## Introduction

Podocytes are highly differentiated kidney cells that produce the slit diaphragm, a key component of the renal glomerulus filtration barrier, responsible for removing toxins and metabolic waste while retaining leukocytes and larger proteins in the bloodstream. Podocytes also contribute significantly to the formation of the glomerular basement membrane and the integrity of the glomerular microvascular endothelium. Thus, podocyte injury and/or loss of podocytes leads to impaired blood filtration and is the cause of many common renal diseases characterized by severe proteinuria (the leakage of serum proteins into the urine) and hypoalbuminemia (low serum albumin levels). Continued injury to podocytes can lead to irreparable damage to the glomerulus and kidney function resulting in renal failure.

As in many organs, podocyte injury repair partially recapitulates fetal developmental processes. Critical to podocyte development and differentiation are events mediated by numerous cell permeable hormones [1]. These small lipopholic molecules such as steroids, fatty acids, prostaglandins and vitamin metabolites control many aspects of animal development through binding to a family of intracellular receptors, the nuclear receptors (NRs). Upon ligand binding, NRs switch on or off an array of gene networks. The ability of small molecule hormones to regulate NR activity makes them excellent pharmaceutical targets. In addition to normal development, clinical evidence and animal studies have implicated several NRs in podocyte diseases [2]. Recent studies from animals and cultured human or mouse podocytes indicate that synthetic hormones including ligands for estrogen receptor (estradiol), glucocorticoid receptor (glucocorticoid), retinoid receptors (retinoid), vitamin D3 receptor (vitamin D3), and peroxisome proliferator-activated receptor alpha (pioglitazone and WY-14643) protect or rescue podocytes from experimental glomerular injury [3], [4], [5], [6], [7], [8], [9]. Nonetheless, the mechanism underlying the ability of these hormones to protect podocytes and kidney function is an important issue for patient treatment that remains poorly understood. This is partly due to the limited knowledge of the target genes and affected pathways controlled by these hormones. In addition, glucocorticoid treatment studies in animals and patients cannot separate direct versus indirect effects, thus more comprehensive studies should provide valuable information on the direct effects on the renal cells, the intended target of the therapy.

In addition, recent patient and animal studies have suggested that vitamin D also may provide beneficial renoprotective functions, but the therapeutic utility of vitamin D treatment for common diseases such as diabetic nephropathy remains uncertain [10], [11]. In order to elucidate the mechanisms by which glucocorticoids and Vitamin D3 elicit their renoprotective activity, we initiated a gene expression profiling study to identify their target genes and affected pathways in cultured human podocytes. Immortalized human podocytes (HPCs) were induced to differentiate followed by exposure to dexamethasone (Dex) or vitamin D3 (VD3). We found that while Dex and VD3 are known to both protect podocytes from experimental injury, their effects on gene expression and the affected pathways were quite different. Dex appears to alter more target genes than VD3 and thus affects a broader spectrum of signaling pathways. Taken together, our data indicate that these two hormones have limited common gene targets in differentiating podocytes. Furthermore, our study opens new avenues for future investigation into the molecular pathways by which hormones protect podocytes from injury.

## Materials and Methods

### Cell Cultures, *in vitro* Differentiation, Hormone Treatment and Sample Preparation

The human podocyte cell line (HPCs), which has been previously described [12], [13], is considered the standard cell line for podocyte experiments, and *in vitro* differentiation of HPCs was carried out as described [12]. Briefly, temperature-sensitive HPCs were maintained in culture medium containing RPMI supplemented with 10% charcoal stripped fetal bovine serum (FBS), 1% antibiotics and Insulin-Transferrin-Selenium (GIBCO #51500) at the permissive (undifferentiated) temperature of 33°C. For differentiation, HPCs at 70–80% confluence were transferred to a cell culture incubator set to the non-permissive (differentiation) temperature of 37°C for 2 days followed by DMSO (Veh.), 100 nM dexamethasone (Dex), or 100 nM vitamin D3 (VD3) treatment for 3 more days at 37°C. Undifferentiated HPCs were treated with vehicle for 3 days at 33°C. Total RNA was isolated, prepared and processed as described elsewhere [14]. For time-course experiments, differentiating HPCs were treated with vehicle or 100 nM of Dex or VD3 for 2, 8, 24, 48 or 72 h. For dose-dependent experiments, differentiating HPCs were treated with 10 nM, 100 nM or 1 µM of Dex or VD3 or with vehicle for 4 or 72 h.

### Immunofluorescence Microscopy

HPCs were plated onto coverslips of 12-well plates, grown and treated with or without hormones following differentiation as described above. Fluorescent microscopy was conducted according to our published protocol [15]. Briefly, cells were washed with 1X PBS, fixed in 3.7% paraformaldehyde for 30 min at room temperature and incubated with rhodamine-labeled phalloidin (Cytoskeleton, cat. # PHDR1). DAPI was applied to the samples after the final wash to visualize cell nuclei. Images were visualized using a LEICA fluorescence microscope equipped with a camera.

### Microarray Experimental Design, Data Pre-processing and Gene List Retrieval

The microarray was carried out on the illumina beadarray platform with HumanRef-8 chip (V3_0_R3_11282963). Following a fractional factorial design, the microarray study was to examine the effects of two factors: (i) Differentiation and (ii) Hormone effects during differentiation. The differentiation factor has two levels (Differentiated and Undifferentiated) while the hormone factor has three levels (Veh, VD3 and Dex). The raw data with background noise removed was analyzed in the R/Bioconductor environment [16], [17] as previously described [14]. Briefly, the raw data were pre-processed with the following packages: lumi [18], [19], VST [20], RSN [20], and LIMMA [21]. During these processes, the probes with non-significant detection p-values (p>0.1) in all samples were removed from the analysis. The pre-processed expression data were fit to a general linear model adjusted by the empirical Bayes method and the false discovery rate (FDR) was adjusted by the Benjamini and Hochberg’s method. The significantly changed gene lists were then complied (>1.5 fold and FDR adjusted p (q) <0.05). For functional analyses, the most significantly changed (>2 fold and q<0.01) gene lists were used. The microarray data has been deposited to GEO database with accession number GSE39823.

### Functional Ontology Analyses

The 3-D terrain sample relationship analysis, clustering with gene ontology (GO), Kyoto Encyclopedia of Genes and Genomes (KEGG) and the k-means clustering were performed in the MultiExperiment Viewer (MeV v4.8.1, an R-based software) [22]. Additional hierarchical clustering was analyzed in R with average linkage. The sub-clusters were identified in the dynamic TreeCut R package [23]. The functional annotation enrichment tests were carried out in the Database for Annotation, Visualization and Integrated Discovery (DAVID, v6.7, http://david.abcc.ncifcrf.gov/) [24]. The venn diagram was plotted with Venny (http://bioinfogp.cnb.csic.es/tools/venny/index.html) [25]. The module-trait correlation and module identification studies were done in the WGCNA R package [26]. The functional analysis of transcriptional networks was analyzed by FUNNET [27] and visualized in Cytoscape [28] software. The customized Entrez keyword enrichment analysis and tabular heatmaps were done with the GeneAnswers R package [29]. Details of each analysis are described in the *Supporting methods and materials* (Methods S1).

### Total RNA Extraction, RT-PCR, and Real-time PCR

Total RNA was extracted using a PrepEase kit (USB/Affymetrix) according to manufacture’s protocol (USB) and quantified by A260/A280 spectrometry. The cDNA pool was synthesized from 1 µg of total RNA with Superscript3 Reverse Transcriptase according to the manufacturer’s instructions (Invitrogen). qRT-PCR was conducted using an iCycler (Bio-Rad) platform with 2_iQ SYBR Green Supermix (Bio-Rad) with the gene-specific primers (Table S6). The PCR program was set for 40 cycles with three steps of 95°C for 10 s, 55°C for 20 s, and 72°C for 30 s. Melting curves were acquired after PCR to ensure the homogeneity of the PCR products. The relative levels in gene expression were normalized to an internal control, *GAPDH* and calculated using the ΔΔCt method. Each value is representative of three replicates and all the experiments were repeated twice. The primer sequences are enclosed in Table S7.

### Western Blotting

Vehicle, Dex or VD3-treated HPCs were harvested day 1, 2 or 3 days after treatment, whole cell extracts prepared and Western blotted with anti-GRα (Santa Cruz, sc-8992), anti-VDR (Santa Cruz, sc-1008) or anti-β-actin (Sigma-Aldrich, A-5441) antibodies.

## Results

### Hormone Treatment Modulates the Transcriptome during HPC Differentiation

**Figure 1:**
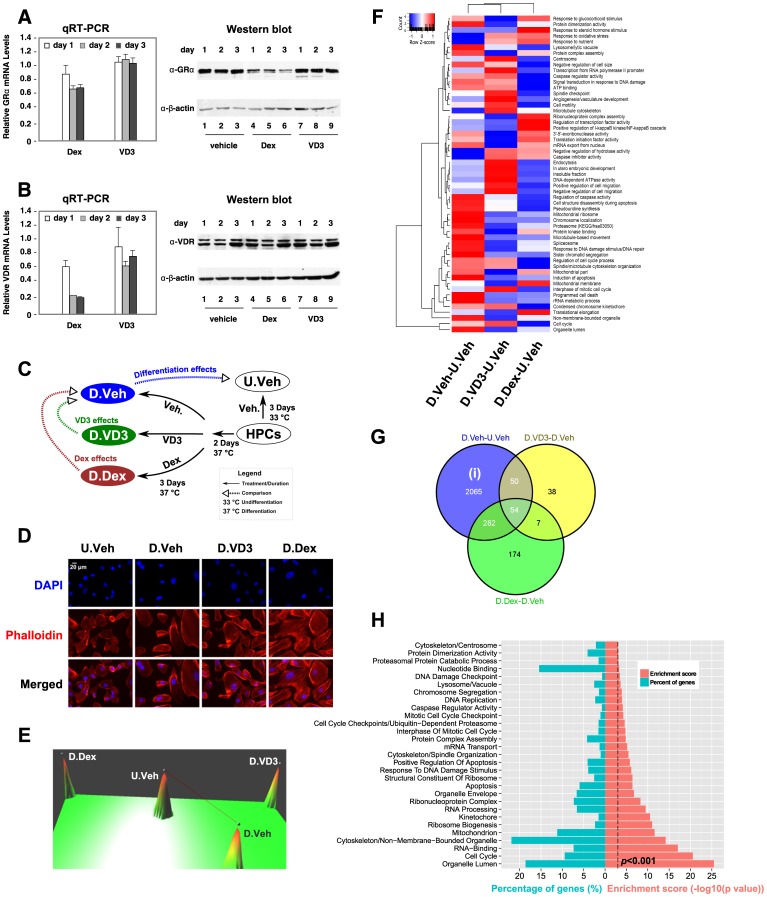
Microarray analysis of HPC differentiation. ***A*–*B***, HPCs were induced to differentiation according to a published protocol [12]. Two days after induction, cells were treated with vehicle, Dex or VD3. HPCs were harvested daily and total RNA was isolated and the accumulations of mRNA and protein of GRα (***A***) and VDR (***B***) were determined by qRT-PCR and Western blots, respectively. The relative mRNA expression is normalized by vehicle-treated samples. The β-actin was used as an internal Western loading control. (**C**) A schematic representation of the experimental design for microarray study. (**D**) The effects of hormone treatment on the morphology of differentiated HPCs. HPCs were treated as described in (***C***) followed by staining with rhodamine-labeled phalloidin and DAPI and visualized with fluorescent microcopy as described in *Methods*. Scale bar represents 20 µm. (**E**) A 3-D gene expression terrain map [52] of the sample relationship based on the significantly changed probes (>1.5 fold and *q*<0.05). The samples are represented by peaks and the correlation between samples is linked by lines with a correlation efficiency cutoff of 0.9. (**F**) Relative enrichment scores of the significantly influenced functional ontology information (*P<*0.01 in at least one of the comparisons) during HPC differentiation after vehicle or hormone treatments. (**G**) Comparison of the membership of the significantly changed genes (>1.5 fold, q<0.05). Genes (2065) representing the hormone-independent differentiation effects were designated as (i). The gene lists, D.Dex-D.Veh and D.VD3-D.Veh, represent the hormonal effects during HPC differentiation. (**H**) Significantly influenced functional categories (*P<*0.001) based on genes in (i) analyzed by DAVID as described in *Methods*. The percentage of genes (%) in the specific categories is also shown.

Immortalized human podocytes (HPCs) were maintained in culture medium at 33°C and induced to differentiate at 37°C. We first examined the expression levels of GRα and VDR by qRT-PCR using two pairs of primers for both GRα and VDR. We found that both GRα and VDR were expressed in HPCs, but that GRα mRNA levels were expressed 30-fold higher than VDR (data not shown). While Dex treatment reduced GRα and VDR mRNA levels, VD3 treatment does not affect GRα mRNA accumulation but modestly decreased VDR mRNAs (Figure 1A–B). At the protein levels, Dex treatment decreased GRα protein accumulation after one day of treatment, but did not affect VDR protein levels (Figure 1A). By contrast, VD3 had little or no effects on neither GRα nor VDR protein expression (Figure 1B). We found that during induction of differentiation, these cells gradually stop proliferating and start differentiating and this transition takes approximately two days. Because injured podocytes in vivo undergo similar dedifferentiation processes [12], we reason that treated differentiating HPCs with hormones may mimic the effects of hormones on injured podocytes. To examine the optimal hormonal effects during the differentiation process, two days after induction, HPCs were treated with vehicle, 100 nM vitamin D3 (VD3) or 100 nM dexamethasone (Dex) for 3 more days (Figure 1C). Cells were harvested; total RNA was prepared and subjected to microarray analysis. The experimental design defined 4 different conditions of samples: undifferentiated (33°C) HPCs treated with vehicle (U.Veh), differentiated (37°C) HPCs treated with vehicle (D.Veh) or hormones (D.VD3 and D.Dex) (Figure 1C). The effects of Dex and VD3 on the morphology of differentiating HPCs were further visualized by immunofluorescent microscopy (Figure 1D). Consistent with a previous report [12], differentiated HPCs were morphologically distinct (larger in size) compared to undifferentiated HPCs. Furthermore, Dex treated differentiated HPCs exhibit a substantial increase in stress fibers and filopodia compared to vehicle treated HPCs, whereas vehicle or VD3 treated HPCs showed only modest increases in stress fibers and filopodia (Figure 1D).

Comparing the profile of probe densities from raw scale data with those from the transformed and normalized data, we found that the profiles of the transformed and normalized data are well overlapped to each other (Figure S1). The technical duplicates are closely aligned to each other in the sample dendrogram (Figure S2A) and show small variation within inter-quantile range (IQR) (Figure S2B). These data indicate a good quality control of our transformation and normalization procedure and establish a basis for comparison between samples. The sample relationship was visualized in a 3-D terrain expression map (Figure 1E). We found that the 4 different conditions were well separated and that the Dex-treated differentiated sample (D.Dex) and the VD3-treated differentiated sample (D.VD3) were the most distantly separated compared to the vehicle-treated differentiated sample (D.Veh) (Figure 1E). We first compared the HPC differentiation processes under vehicle treatment (D.Veh-U.Veh) to VD3 treatment (D.VD3-U.Veh) or Dex treatment (D.Dex-U.Veh). The significantly influenced ontology information (*P<*0.01) in any of these differentiation processes were analyzed and exported using DAVID bioinformatic suites [24]. The enrichment scores are presented as relatively enriched (positive Z-score) or relatively suppressed (negative Z-score) as previously described [30] (Figure 1F). Thus, the relative influences of Dex or VD3 hormone treatment on different biological processes during the HPC differentiation can be visually presented (Figure 1F).

The significantly changed genes (>1.5 fold, *q*<0.05) were retrieved and annotated (Table S1). The expression profile of these genes across the different conditions was clustered and displayed in a heatmap (Figure S3). We are particularly interested in differentiation effects (D.Veh-U.Veh) and the hormonal effects on differentiation: VD3 effects (D.VD3-D.Veh) and Dex effects (D.Dex-D.Veh). These hormonal effects were retrieved as D.VD3-D.Veh = (D.VD3-U.Veh)-(D.Veh-U.Veh) and D.Dex-D.Veh = (D.Dex-U.Veh)-(D.Veh-U.Veh). The membership comparisons between these gene lists are summarized in a Venn diagram (Figure 1G). We designated the genes in the cluster (i) as differentiation-specific genes independent of hormonal effects and the gene list was enclosed in Table S1. Using DAVID bioinformatics suites, the significantly influenced (*P<*0.001) functional categories based on GO and KEGG databases are summarized in Figure 1H. Our analysis revealed that HPC differentiation is a multifaceted process involving changes in *cell cycle, organelle-related, cytoskeleton, mitochondrion, ribosome biogenesis, apoptosis and lysosome-associated genes*, which collectively direct the dramatic morphological changes observed during differentiation.

### Functional Analysis of Hormonal Effects on Differentiating HPCs

**Figure 2:**
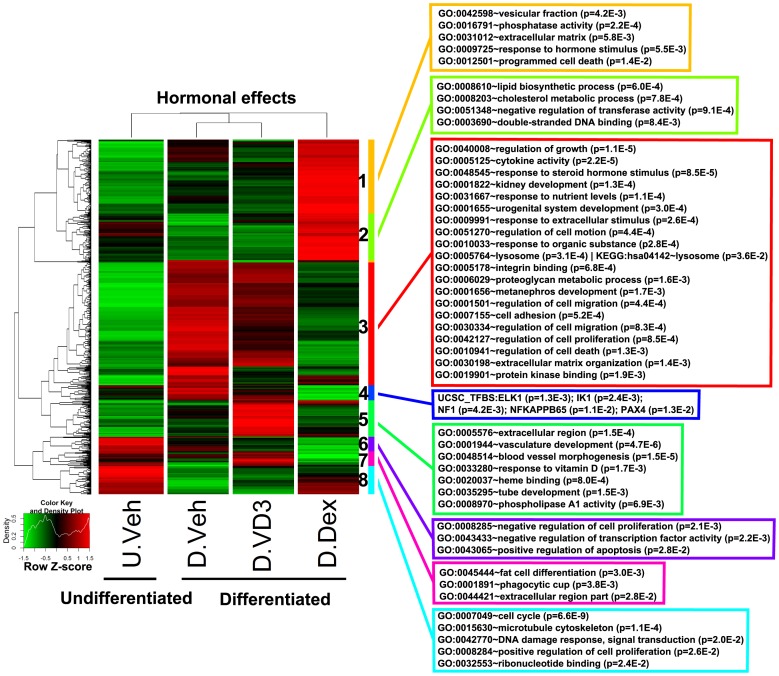
Clustering and functional analysis of genes representing hormonal effects during HPC differentiation. The genes representing hormonal effects (D.Dex-D.Veh and D.VD3-D.Veh) were clustered and their relative expression was plotted using a heatmap. The subclusters were identified as described in *Methods* and labeled by the color sidebar and numbers (1–8). Functional analysis identified the significantly influenced categories (*P<0.05*) associated with each subcluster and the representative ontology terms are shown in the color-matched boxes.

We further focused our study on the hormonal effects (D.Dex-D.Veh and D.VD3-D.Veh) on gene expression in differentiating HPCs. To analyze the categories of genes affected by the hormonal effects, we performed a clustering analysis of these hormonal specific genes and identified several sub-clusters [23] (Figure 2). Through analysis with DAVID bioinformatics suites, we identified significantly enriched (*P<*0.05) functional categories based on multiple ontology databases through DAVID. The identifiers, description of the categories and the associated P values are listed in the colored boxes matched to the annotated side bars in Figure 2. The full lists of the genes and the enriched functional categories in each sub-cluster are listed in Table S2.

**Figure 3:**
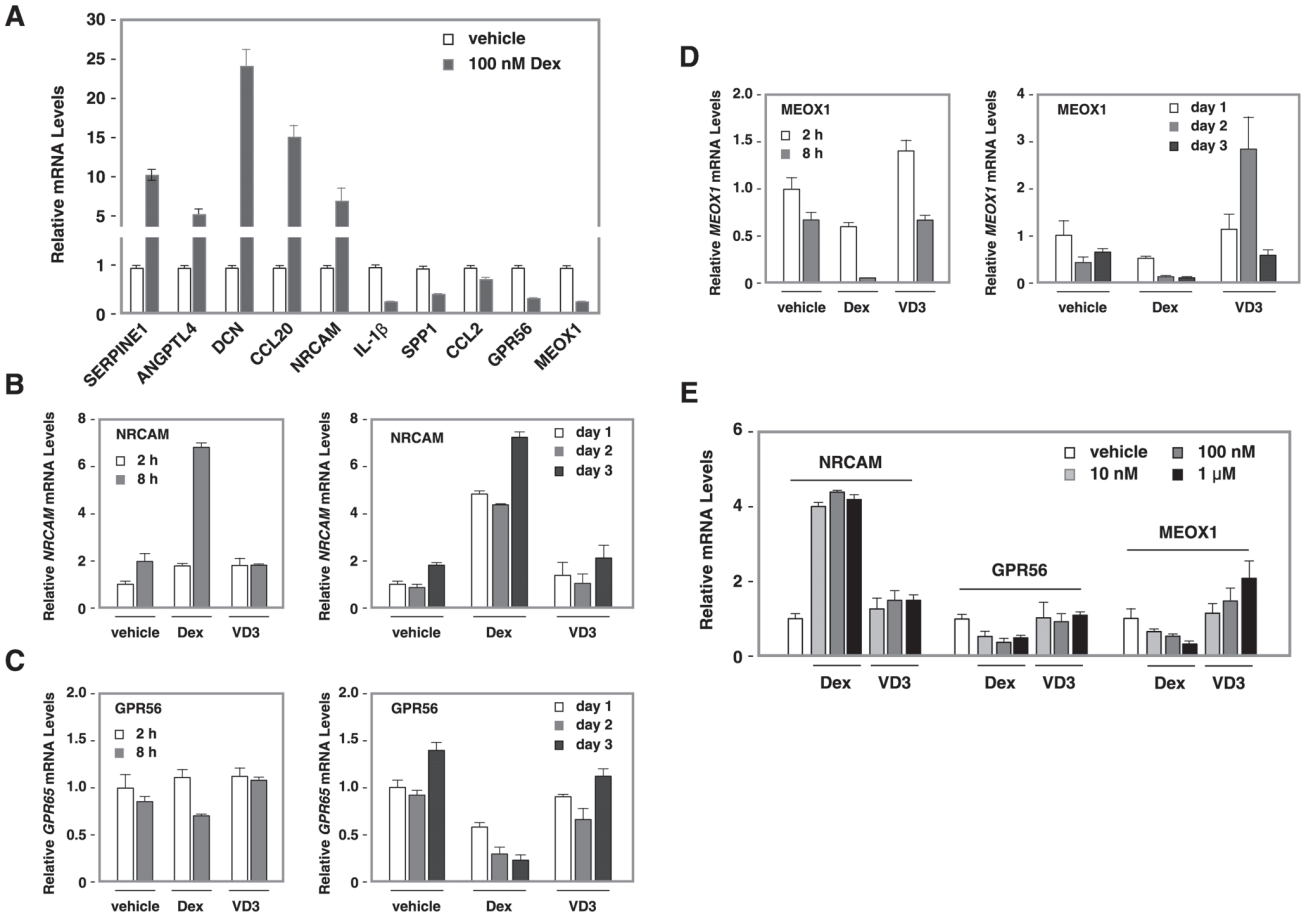
The effects of Dex on NRCAM, GPR56 and MEOX1 mRNA accumulation. (**A**). Top five genes that were up-regulated and top five genes that were down-regulated by Dex in HPCs. In vitro differentiation of HPCs, total RNA isolation and qRT-PCR were described in ‘Materials and Methods. The relative mRNA levels of [+Dex]/[-Dex] are shown for each gene. (**B–D**). A time-course experiment of Dex’s effects on *NRACM*, *GPR56* and *MEOX1* mRNA levels. HPCs were induced to differentiation for two days followed by hormone treatment. Cells were harvested 2, 8, 24, 48 and 72 h after treatment, total RNA isolated and qRT-PCR performed. The relative mRNA levels of *NRCAM* (**B**), *GPR56* (**C**) and *MEOX1* (**D**) are normalized by GAPDH mRNA and the value at 2 h (left) or day 1 (right) is set at 1. (**E**) Dosage-dependent responses of NRCAM, GPR56 and MEOX1 mRNAs to Dex treatment. Differentiating HPCs were treated with vehicle, 10 nM, 100 nM or 1 µM of Dex or VD3, cell harvested 4 h after treatment, total RNA prepared and subjected to qRT-PCR.

Focusing on Dex treatment effects on differentiation, we validated the microarray data regarding the effects of Dex on the expression of the top 5 genes that were induced or down-regulated by Dex treatment by qRT-PCR. Figure 3A shows the effects of Dex on the expression of the top 5 up-regulated and the top 5 down-regulated genes following Dex treatment. Their expression patterns are consistent with our microarray data. Among these genes, proteins encoded by *SERPINE1* [*31]*, [32], *ANGPT4* [*33]*, [34], *DCN* [*35*], *CCL20* [*36]*, [37], *IL-1β* [*38]*, [39], *SPP1* [*40]*, [41] and *CCL2* [*42]*, [43] have previously been described to play roles in kidney development or renal disorders including focal segmental glomerulosclerosis (FSGS). By contrast, the role of proteins encoded by *NRCAM*, *GPR56* and *MEOX1* have not been directly linked to podocyte function. We further performed time course experiments on these three genes. Dex induced 2 fold *NRCAM* mRNA accumulation 2 hours after Dex treatment and this induction was sustained for three days (Figure 3B–D). Similarly, we observed a decrease in *GPR56* and *MEOX1* mRNA levels 2 hours after Dex treatment. However, VD3 had little or no effect on the expression of these three genes. Four hours after treatment, 10 nM of Dex showed the same potency as 1 µM of Dex to induce *NRCAM* expression. Furthermore, Dex exhibited slight dosage-dependent repression on *GPR56* and *MEOX1*. However, VD3 had little effects on *NRCAM*, *GPR56*, or *MEOX1* expression (Figure 3E).

**Figure 4:**
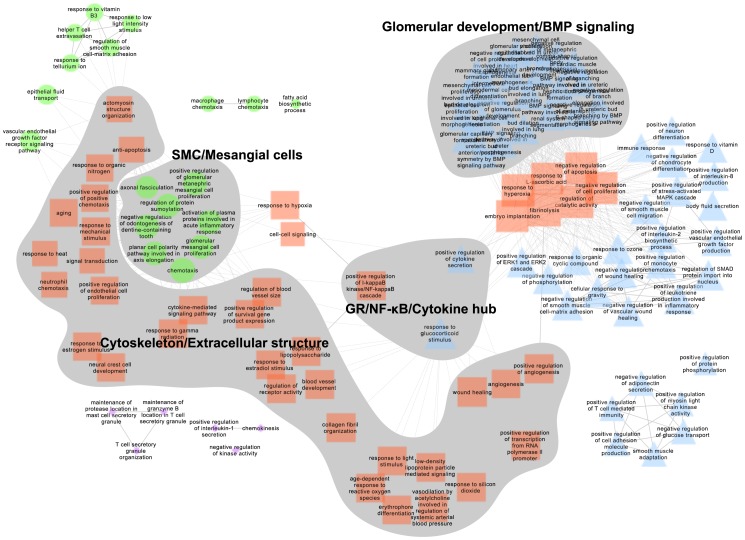
GO.BP functional annotation co-expression network of Dex effects. The GO.BP annotation network was analyzed by FUNNET and visualized by Cytoscape using the most significantly changed genes (>2 fold, q<0.01) from the gene list D.Dex-D.Veh. The different shapes and colors of the nodes represent membership of distinct network modules identified by FUNNET analysis. The size of node correlates with the functional centrality of the network. GO.BP terms are labeled on the node. Solid line, intra-modular link; dashed line, inter-modular link. Shading marks interesting portions of the network that correlate with specific functions.

We further carried out analysis of the enriched gene networks after Dex treatment (D.Dex-D.Veh gene list, Table S1) using FUNNET [27]. Employing the enriched terms from GO.BP, we identified several interesting network nodes (Figure 4), including those involved in processes related to ”Glomerular development/BMP signaling”, ”Cytoskeleton/Extracellular structure” and ”Smooth muscle cell/Mesangial cells”. For better visual ability of the network, closely related terms are placed in a stacked fashion. The full lists of the significantly influenced ontology terms (Table S3) and the genes associated with the specific terms (Table S4) are reported in the Supporting tables. Interestingly, we also identified networks participating in the response to glucocorticoid stimulus and regulation of the IκB/NF-κB cascade and cytokine secretion (Figure 4). The co-expression network of the genes representing these GO.BP ontology terms are shown in Figure S4. The co-expression networks of Gene Ontology Cellular Component (GO.CC), GO.MF and KEGG are shown in Figure S5.

**Figure 5:**
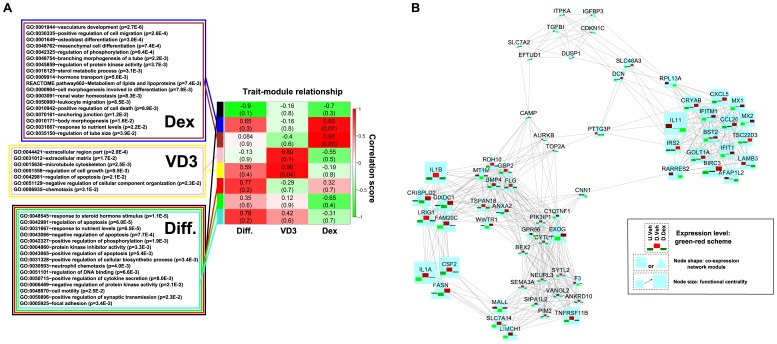
Modules associated with hormonal treatments during HPC differentiation identified by weighted gene co-expression network analysis. The significantly changed probes (>1.5 fold, q<0.05) representing hormonal effects (D.Dex-D.Veh and D.VD3-D.Veh) were used for the WGCNA analysis. (**A**) Three traits of the samples were defined to study the module-trait relationships: ''Diff'' trait categorizes the samples into differentiated (D.Veh, D.VD3, D.Dex) and un-differentiated (U.Veh). “VD3” trait categorizes the samples into untreated (U.Veh, D.Veh. D.Dex) or VD3-treated (D.VD3). ''Dex'' trait categorizes the samples into untreated (U.Veh, D.Veh, D.VD3,) or Dex-treated (D.Dex). The module-trait correlation is represented as a heatmap with correlation coefficient (r) scaled to a green-white-red scheme. The correlation coefficients and the *p* values are shown in each box. Specifically, the blue and brown modules are highly correlated with the “Dex” trait. The pink and yellow modules are highly correlated to ''VD3'' trait. The rest of modules are modestly or weakly correlated with the “Diff” trait. Functional analysis identified significantly (*P<*0.05) influenced functional categories for different trait-associated modules. The ontology terms are listed in the color-matched boxes. (**B**) The co-expression network of the most significantly changed genes (>2 fold, q<0.01) that are highly correlated with the “Dex” trait. The network was analyzed by FUNNET and visualized in Cytoscape. The scaled expression values of each gene under the different conditions (U.Veh, D.Veh, and D.Dex from left to right) were overlaid on the node with a green-red scheme [53]. Node shape reflects membership of different network modules. The node size is scaled to the functional centrality.

To identify the signature genes that correlate with the hormone treatment, we carried out a scale-free topological co-expression network analysis using hormone-specific genes through the WGCNA R package [26] (Figure 5). In a topological heatmap representation of the hormonal specific gene network, the gene signatures/modules were evident as squares in red along the diagonal (Figure S6). Therefore we identified several modules that are annotated by the color side bars (Figure S6). The genes in each module and the related annotation information are listed in Table S5. These identified gene signatures/modules were further analyzed for their correlation with the phenotypic traits of the samples. The samples can be categorized into different groups based on three different traits: differentiation, Dex treatment, and VD3 treatment. The correlations between modules and traits are presented quantitatively in a heatmap (Figure 5A). We found that the sample trait VD3 treatment is strongly correlated with the pink and yellow modules, while the Dex treatment was strongly correlated with the blue and brown modules. The black module was negatively correlated with the differentiation trait and the rest of the modules (red, green, and turquoise) were modestly or weakly correlated with differentiation. For these modules, through DAVID analysis [24], we identified the significantly enriched functional categories that are listed in the color-matched boxes in Figure 5A. Similar to the analysis in Figure S4, we visualized the most significantly changed genes (>2 fold, *q*<0.01) from the Dex-correlated modules (blue and brown) as co-expression weighted networks [26], [27] in Figure 5B.

**Figure 6:**
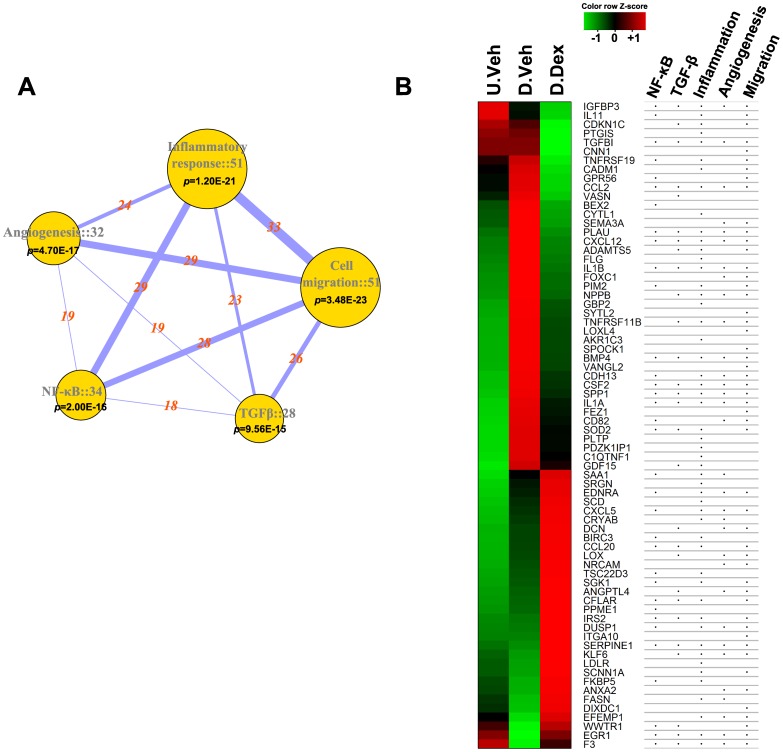
Entrez keyword-based functional annotation analysis unveils unique effects of Dex treatment during HPC differentiation. The most significantly changed probes (>2 folds and *q*<0.01) were used for the analysis. The Entrez keywords tested were: Inflammatory response, Cell migration, Angiogenesis, NF-κB and TGFβ. The gene networks associated with these keywords were retrieved from NCBI Entrez database [29]. (**A**) The gene networks of the tested keywords are summarized. The number of significantly changed genes and the p values are shown with the category nodes. The node size is proportional to gene number. The numbers of common genes shared by different category nodes are marked with the connecting lines. The width of line is proportional to the number. (**B**) Dex effects on the gene expression during HPC differentiation. The genes were annotated by the different functional categories.

In agreement with the analysis using the D.Dex-D.Veh gene list in Figure S4, the key genes including IL1A, IL1B, CSF2, FASN, TNFRSF11B and IL11 are also shown in the co-expression network of the Dex-correlated modules (Figure 3B). The annotated ontology networks of the Dex treatment trait-correlated modules are shown in Figure S7A. We found that several nodes in the co-expression network show converged biological processes related to HPC differentiation: Glomerular development, Morphogenesis and Inflammatory responses (Figure S7A). Similar to the analysis of GO.BP co-expression network using D.Dex-D.Veh gene list in Figure 4, we also identified a hub of NF-κB/Angiogenesis/Wound healing in this Dex-trait associated GO.BP network (Figure S7A). The GO.CC annotation network (Figure S7B) and KEGG network (Figure S7C) are also shown. NF-κB, the inflammatory response and angiogenesis are cellular processes that have not been previously reported and are affected by Dex treatment during HPC differentiation. To further analyze the influence of Dex treatment on these functions and to classify the significantly changed genes into these cellular process categories, we performed a customized Entrez-keyword based enrichment analysis [29] (Figure 6). We found that NF-κB (*P* = 2.00E–16), the inflammatory response (*P* = 1.20E–21), TGFβ (*P* = 9.56E–15), angiogenesis (*P* = 4.70E–17) and cell migration (*P* = 3.48E–23) were significantly affected and highly associated with each other following Dex treatment during differentiation (using the D.Dex-D.Veh most significantly changed gene list, >2 fold, *q*<0.01) (Figure 6A). The gene expression levels from U.Veh, D.Veh and D.Dex are presented in a heatmap with a green-black-red scheme and the corresponding membership of each gene in the above mentioned functional categories are annotated in a table (Figure 6B). Finally, because Dex binds and regulates GR-mediated transcriptional networks, we searched for the potential transcriptional factors whose activity is affected by Dex using the University of California Santa Cruz ENCODE transcription factor binding site database (UCSC_TFBS) through DAVID analysis [24]. We found that NF-κB, AP1 and STATs were among the highest scoring transcription factors that regulate the expression of the identified target genes (D.Dex-D.Veh gene list, the most significantly changed genes, >2 fold, *q*<0.01) (Table S6). Indeed, many Dex-specific target genes in differentiating HPCs (D.Dex-D.Veh) contain the binding sites for NF-κB (65.6%), AP1 (64.7%) and STAT (54.9%) in their promoters (Figure S8).

## Discussion

Synthetic hormones for GR and VDR have been used to treat diverse diseases and as dietary supplements. Podocytes are known to express these receptors, and several *in vitro* studies have demonstrated the importance of glucocorticoids in supporting podocyte survival and optimal phenotype. In this study, we determined the target genes of Dex and VD3 in differentiating cultured HPCs. We have also identified changes in gene expression and affected pathways of HPCs during *in vitro* differentiation. While Dex and VD3 protect podocytes from injury *in vitro* and *in vivo*, our study shows that these hormones affect few common target genes. In fact, our study demonstrates unique responses of HPCs to Dex or VD3 at the molecular level and distinct categories of responding genes. This suggests that these two hormones provide renoprotection through distinct pathways. In supporting this conclusion, qRT-PCR confirmed that Dex and VD3 distinctively regulate the expression of on their target genes (Figures 3B and S8).

Podocyte differentiation is a multi-faceted process involving cell cycle and morphological changes that are driven and guarded by an array of gene transcription alterations. Our *in vitro* model of podocyte differentiation is widely used and well characterized. It employs a conditional immortalization system using ectopic expression of a temperature sensitive SV40 T antigen to enable cell division. During the *in vitro* differentiation process, the T antigen is silenced, removing this stimulus on cell cycle, thus permitting the cells to resume a more typical quiescent state. Although this is an artificial system of differentiation, prior work has established these changes in cell cycle control and expression of cell-specific proteins associated with terminal differentiation proceed similarly to known *in vivo* differentiation processes and patterns [44]. An initial analysis of altered gene expression patterns during differentiation of HPCs revealed the expected changes in key cell cycle regulatory and biogenic processes associated with cell division as well as the expected appearance of podocyte-specific proteins typically associated with podocyte differentiation (Figure 1H). Because our focus here was on hormonal effects, establishing in our system which genes were associated with differentiation was required to assign a physiological significance to the genes associated with hormonal effects versus differentiation effects.

*In vivo*, disease-induced podocyte injury is a process that has been linked with numerous signaling pathways including increased reactive oxygen species (ROS), endoplasmic reticulum (ER) stress, aberrant activation of mammalian target of rapamycin (mTOR), Wnt and TGFβ signaling, activation of small GTPase RhoA, alterations in cytoskeletal proteins and decreased expression of slit diaphragm components [45]. Continued injury or additional oxidative or inflammatory stress leads to chronic pathological and functional changes manifesting as effacement of podocyte foot processes, deterioration of the filtration barrier and overall loss of kidney filtration function, eventually resulting in renal failure. The prior observations that Dex and VD3 protect podocytes from injury suggest that these hormones likely suppress one of the abovementioned disease responses. Glucocorticoid therapy is a mainstay treatment for many nephrotic diseases, and is used mainly for its action in suppressing inflammatory responses mediated by NF-κB signaling. As expected, our data identified several key signaling pathways involving NF-κB-regulated inflammation and inflammatory mediators. This finding is consistent with the well-known role of agonist-bound GR in transrepression of the pro-inflammatory activities of NF-κB and AP-1 transcription factors (Figure S8, [46]). Indeed, our microarray and qRT-PCR data indicated that Dex represses the expression of *IL-1β* and *CCL2*, key NF-κB target genes involved in inflammation.

Our time course experiments indicate that the three newly identified Dex target genes, *NRCAM*, *GPR56* and *MEOX1,* show early responses to Dex treatment (Figure 3B–D), suggesting that these genes are direct targets of Dex. These effects were sustain for 3 days of Dex treatment and were dosage-dependent. ChIP-seq data from the ENCODE project indicate that both *NRCAM* and *GPR56* contain binding sites for GRα, NF-κB and AP-1, while *MEOX1* contain GRα and AP-1 sites in A549 lung cancer [47]. Future experimental confirmation using ChIP analyses will address whether *NRCAM*, *GPR56* and *MEOX1* are GRα direct target genes in podocytes.

Our microarray data produced results consistent with prior gene and protein expression array studies, and also found several genes previously identified in a proteomics analysis of Dex-treated podocytes, including alpha B-crystallin (CRYAB), Annexins (ANXA2), and lysosomal ATPase (ATP6V1B2) [5]. In addition, our microarray study showed that Dex also directly affected several other well-studied pathways in podocytes, such as modestly inducing TGFβ gene expression. As opposed to high levels of paracrine TGFβ stimulation, low levels of autocrine TGFβ signaling in podocytes has been shown to induce G1/G0 arrest and differentiation [48]. This effect may have been further supported by the down-regulation of insulin-like growth factor binding protein 3 [49], a known modulator of TGFβ and its effect on apoptosis, suggesting the TGFβ-IGFBP3 pathway may be an important Dex-induced mechanism to help preserve both cell survival and the fully differentiated, quiescent state of a healthy podocyte. In addition, we found induction of angiogenic factors and other known paracrine signaling molecules important for maintenance of the glomerular microvasculature such as vascular endothelial growth factors (VEGF), Ephrins, and bone morphogenetic proteins (BMPs) that would also support the preservation of normal glomerular function. Interesting, we also observed Dex strongly induced angiopoietin-like 4, a protein known to be a GR-responsive gene, but also one that has been previously associated with proteinuria and nephrotic syndrome [33], [50]. Thus, the induction of Angptl4 may represent a Dex-induced gene associated with the unwanted side effects of glucocorticoid treatment.

The therapeutic use of VD3 therapy for the prevention of chronic kidney disease, especially diabetic nephropathy, has been suggested from several clinical and pre-clinical studies [10], [11]. In our observations of podocyte responses to vitamin D3, we found the direct effects on podocyte gene expression to be less extensive and closely resembles differentiated pattern (Figure 2), and that few of the same pathways affected by Dex overlapped with the VD3 responses (Figure 1F), although VD3 also modestly induced TGFβ. qRT-PCR experiments indicate that VDR mRNA expression is 30 fold lower than GRα in HPCs. It remains possible that the relatively low VDR protein abundance may account for the limited effects of VD3 on gene expression. Of note, some of the changes in gene expression were the opposite for Dex and VD3 (Figure S9). Because the VD3 effects were more limited, one interpretation is that the renoprotective function of VD3 in patients with kidney disease may represent a secondary systemic response to the treatment, in which the renoprotective function is not imparted through a direct effect of VD3 action on the podocyte. By crossing VDR KO mice with transgenic mice expressing podocyte-specific VDR, it was shown that podocyte-specific expression of VDR protects VDR-null mice from severe renal injury [51]. Thus, between these *in vivo* studies and our *in vitro* studies, it is clear that similar to glucocorticoid stimulation, VD3 stimulation can have direct effects on the podocyte.

In summary, our studies have provided an important resource to further understand the important therapeutic benefit of glucocorticoid and vitamin D therapy in the treatment of chronic kidney disease. In comparison to prior studies, a consistent pattern of gene expression to GR and VDR signaling is emerging and will provide a useful experimental resource to understand cell specific responses with systemic treatments.

## Supporting Information

Figure S1 Probe density plot before and after pre-processing raw data.
(PDF)

Figure S2 Sample relationship analysis with the pre-processed data.
(PDF)

Figure S3 Hierarchical cluster of the log scale fold change data by different comparisons.
(PDF)

Figure S4 The function-weighted network of the significantly changed genes by Dex treatment during differentiation (D.Dex-D.Veh).
(PDF)

Figure S5 Networks of the significantly enriched functional annotations for the Dex effects during differentiation (D.Dex-D.Veh).
(PDF)

Figure S6 Specific modules identified associated with Dex or VD3 treatment by weighted gene co-expression network analysis (WGCNA).
(PDF)

Figure S7 Functional weighted networks of the significantly enriched annotations for the Dex treatment associated modules identified by WGCNA analysis.
(PDF)

Figure S8 A profile of the significantly changed genes by Dex treatment during differentiation (D.Dex-D.Veh) regulated by known transcriptional factors.
(PDF)

Figure S9 QRT-PCR validation of the mRNA accumulations of genes following Dex or VD3 treatment.
(PDF)

Table S1 Gene lists (>1.5 fold, q<0.05).
(XLSX)

Table S2 Gene lists for the identified sub-clusters with annotation.
(XLSX)

Table S3 The significantly over-presented Gene Ontology Biological Process (GO.BP) terms associated with Dex effects on differentiation (Gene list D.Dex-D.Veh)
(XLSX)

Table S4 The specific genes associated with each terms reported in Table S3.
(XLSX)

Table S5 Gene lists in the co-expression modules and annotations.
(XLSX)

Table S6 Top predicted transcription factors binding to the Dex-regulated genes during differentiation (D.Dex-D.Veh).
(DOCX)

Table S7 The sequences of primers used in this study.
(DOCX)

Methods S1 All of the detailed methods and materials that are not covered by the main text are included in this supporting file.
(DOCX)
