# Correction: Microarray Analyses of Glucocorticoid and Vitamin D3 Target Genes in Differentiating Cultured Human Podocytes

**DOI:** 10.1371/annotation/ce9027f6-dbf9-4fc9-af40-9e244edf6a6e

**Published:** 2013-06-21

**Authors:** Xiwen Cheng, Xuan Zhao, Simran Khurana, Leslie A. Bruggeman, Hung-Ying Kao

The correct grant number for NCI P20 is CA103736. 

