# Supplementary figures and images for: Microarray Analyses of Glucocorticoid and Vitamin D3 Target Genes in Differentiating Cultured Human Podocytes
Source: PLoS One. 2013 Apr 4;8(4):e60213. doi: 10.1371/journal.pone.0060213 (PMC3617172; doi:10.1371/journal.pone.0060213)

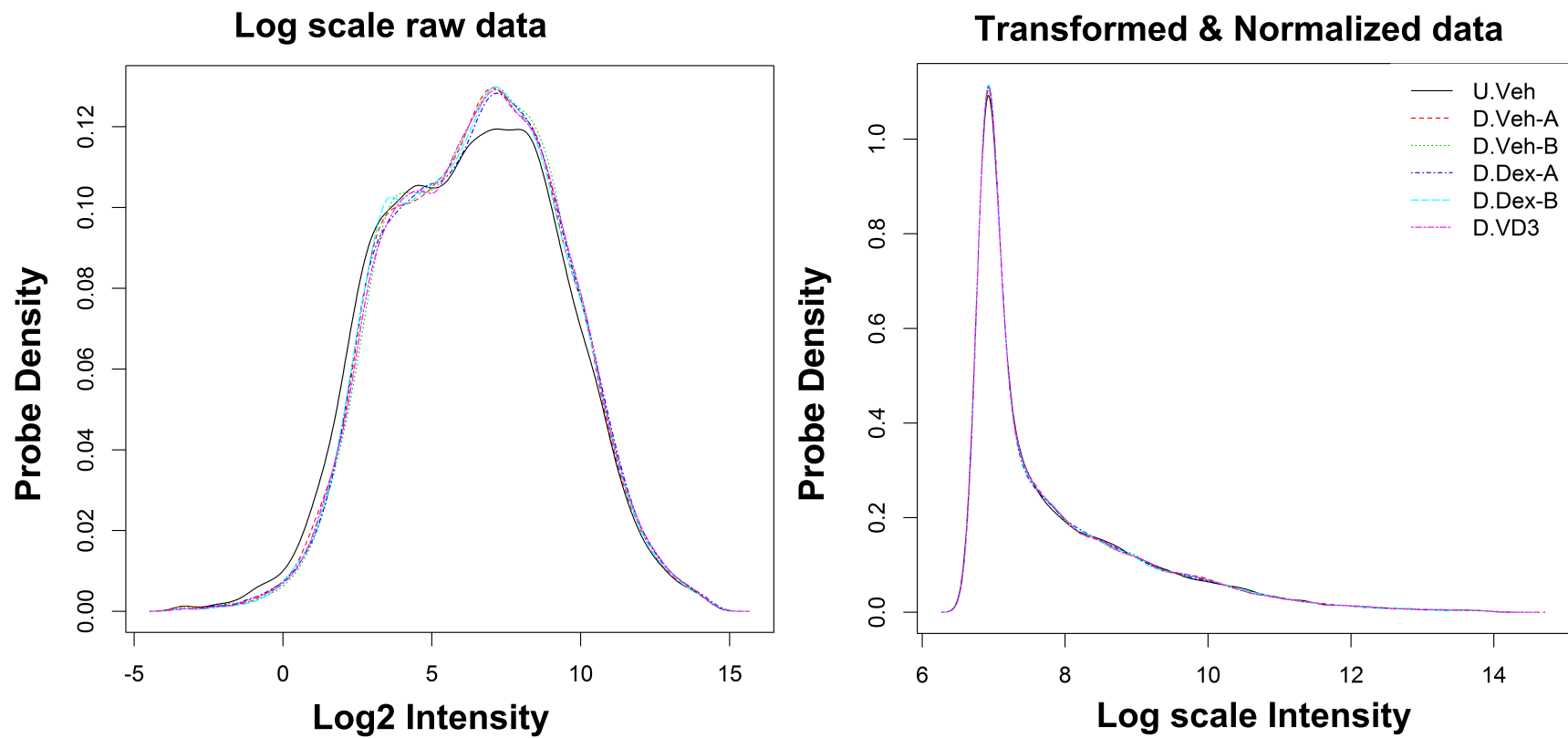

**Figure S1**

Supplement: Figure S1 — Probe density plot before and after pre-processing raw data. (PDF) [file pone.0060213.s001.pdf]

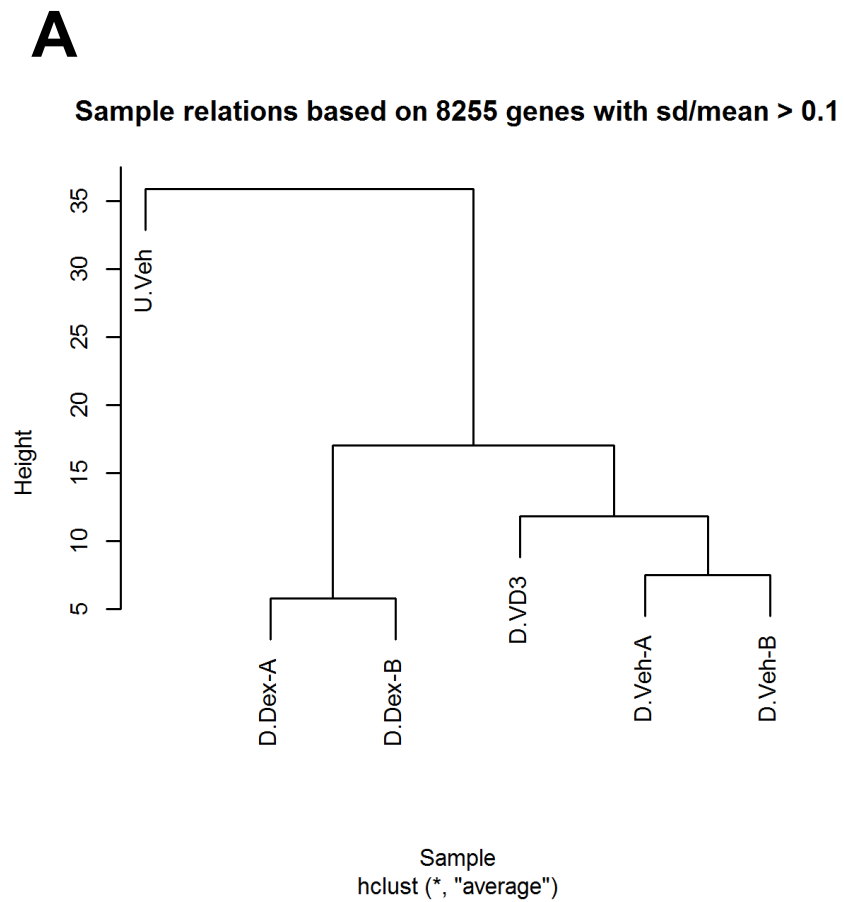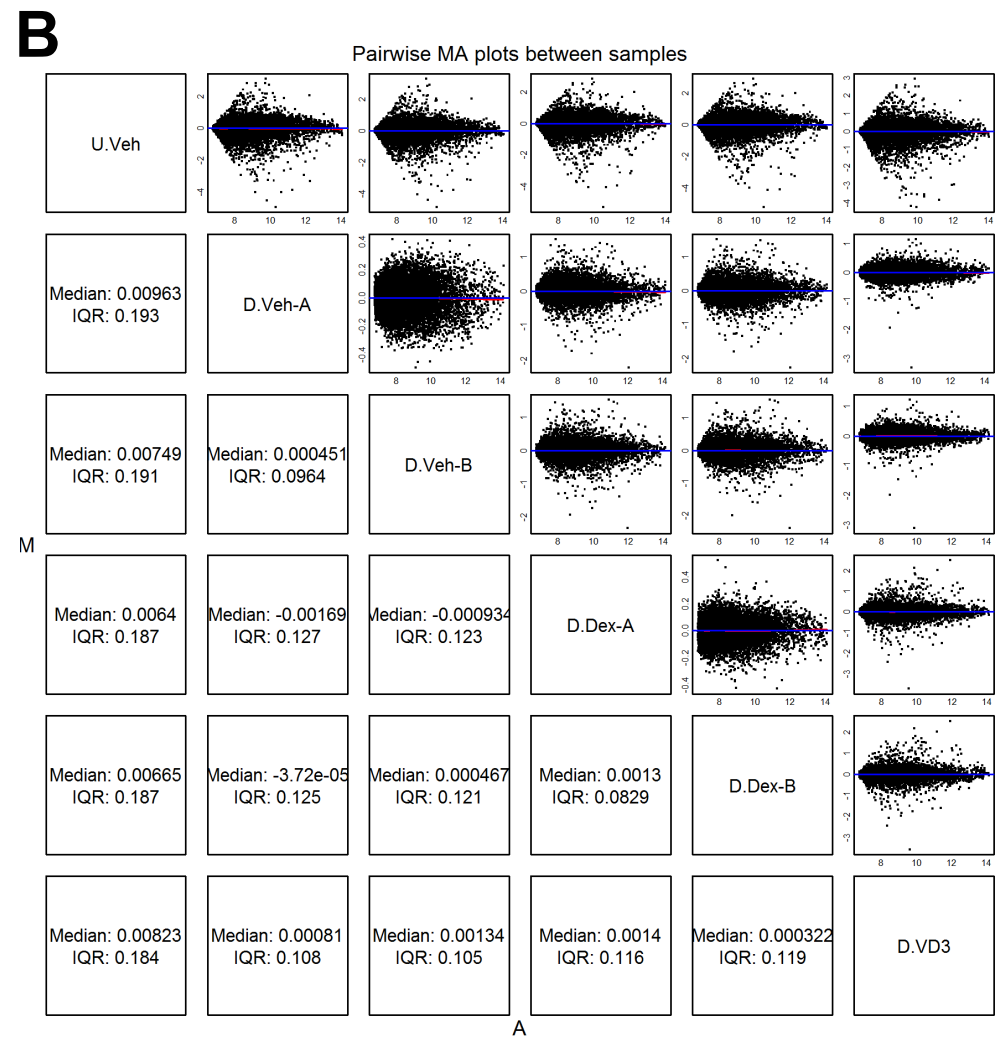

**Figure S2**

Supplement: Figure S2 — Sample relationship analysis with the pre-processed data. (PDF) [file pone.0060213.s002.pdf]

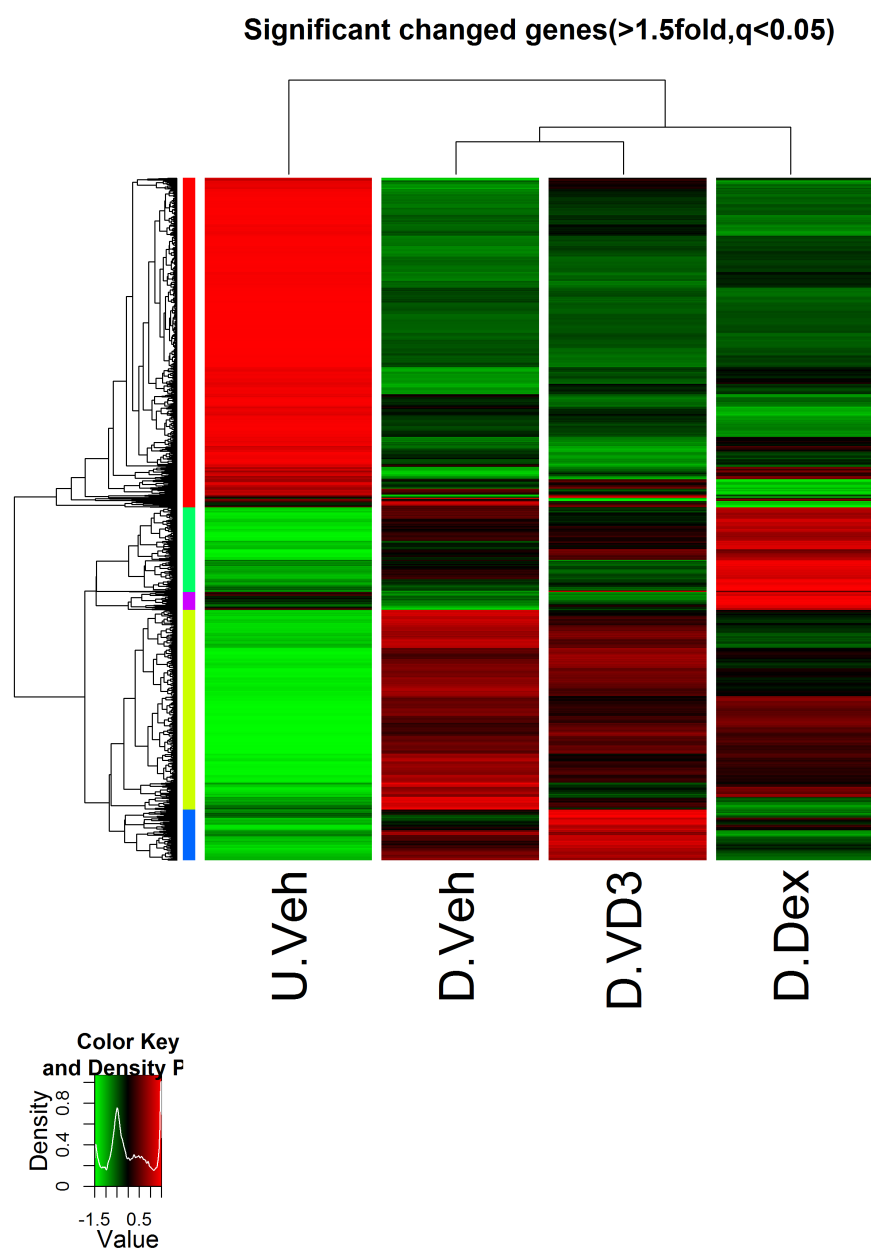

**Figure S3**

Supplement: Figure S3 — Hierarchical cluster of the log scale fold change data by different comparisons. (PDF) [file pone.0060213.s003.pdf]

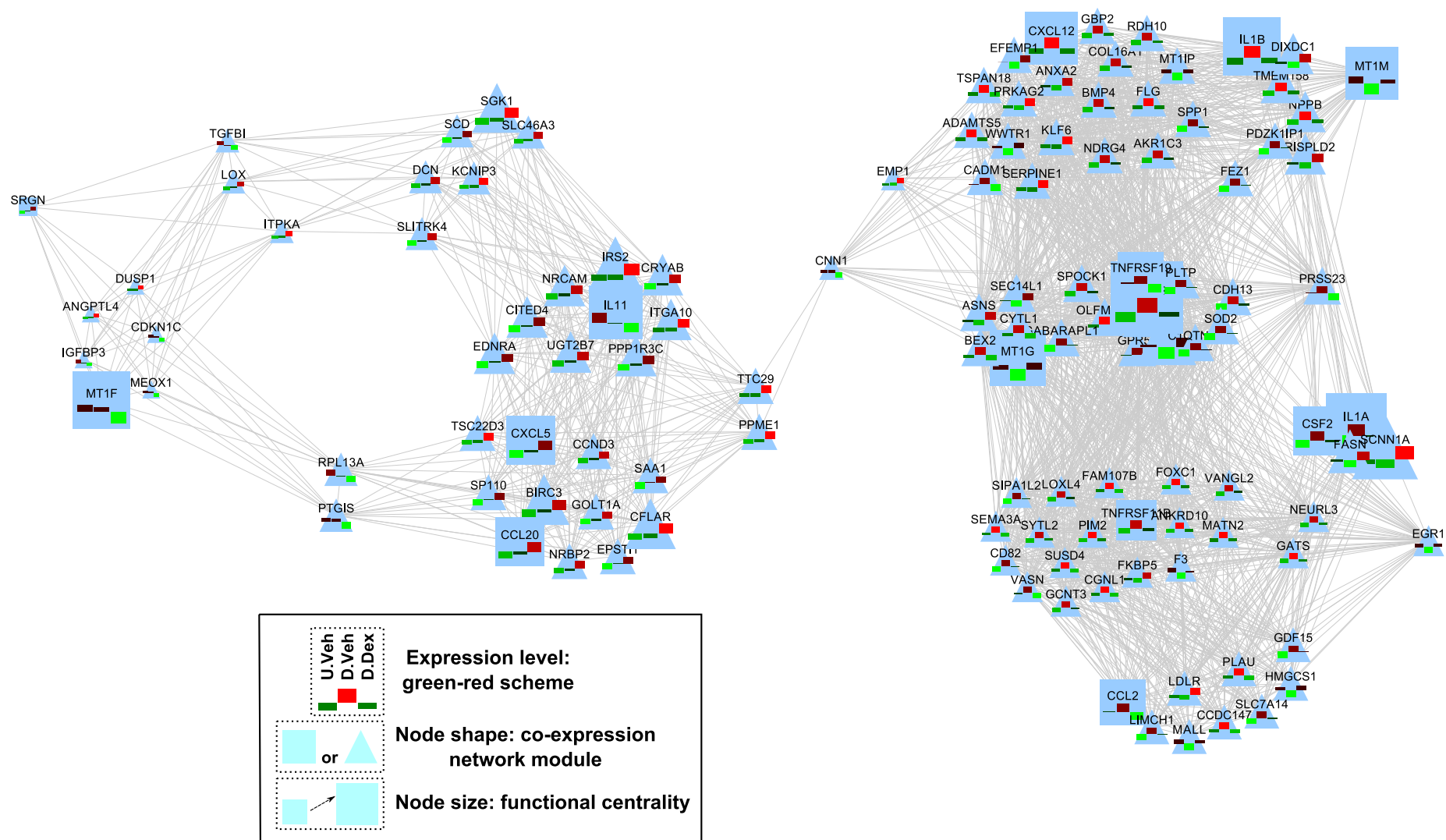

Supplement: Figure S4 — The function-weighted network of the significantly changed genes by Dex treatment during differentiation (D.Dex-D.Veh). (PDF) [file pone.0060213.s004.pdf]

## A. GO.CC annotation network

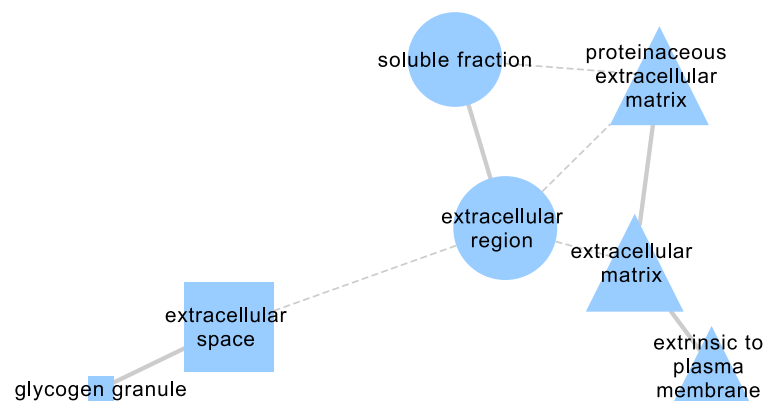

## B. GO.MF annotation network

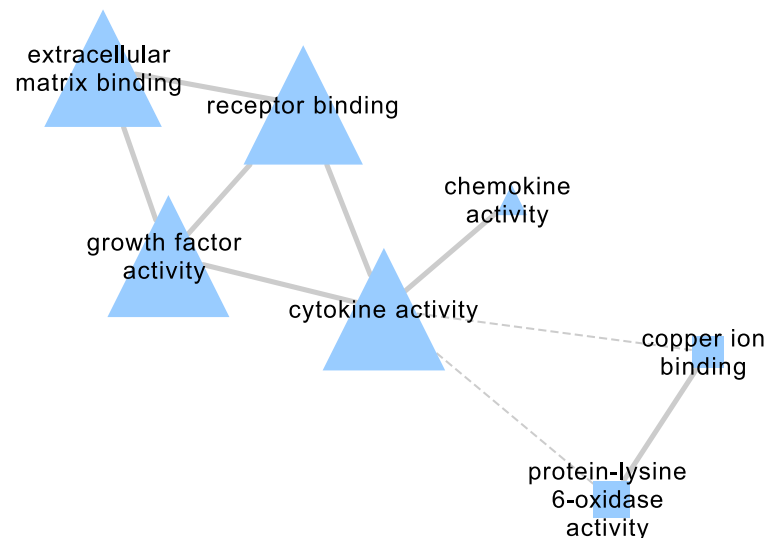

## C. KEGG annotation network

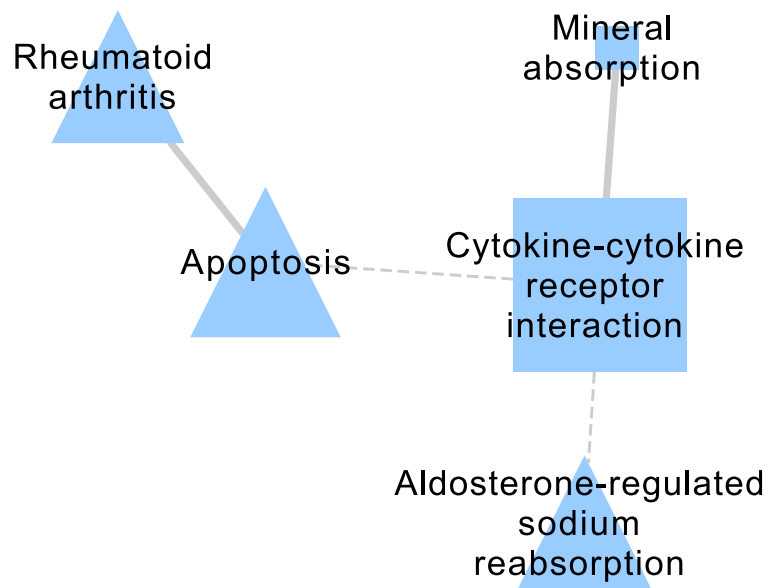

**Figure S5**

Supplement: Figure S5 — Networks of the significantly enriched functional annotations for the Dex effects during differentiation (D.Dex-D.Veh). (PDF) [file pone.0060213.s005.pdf]

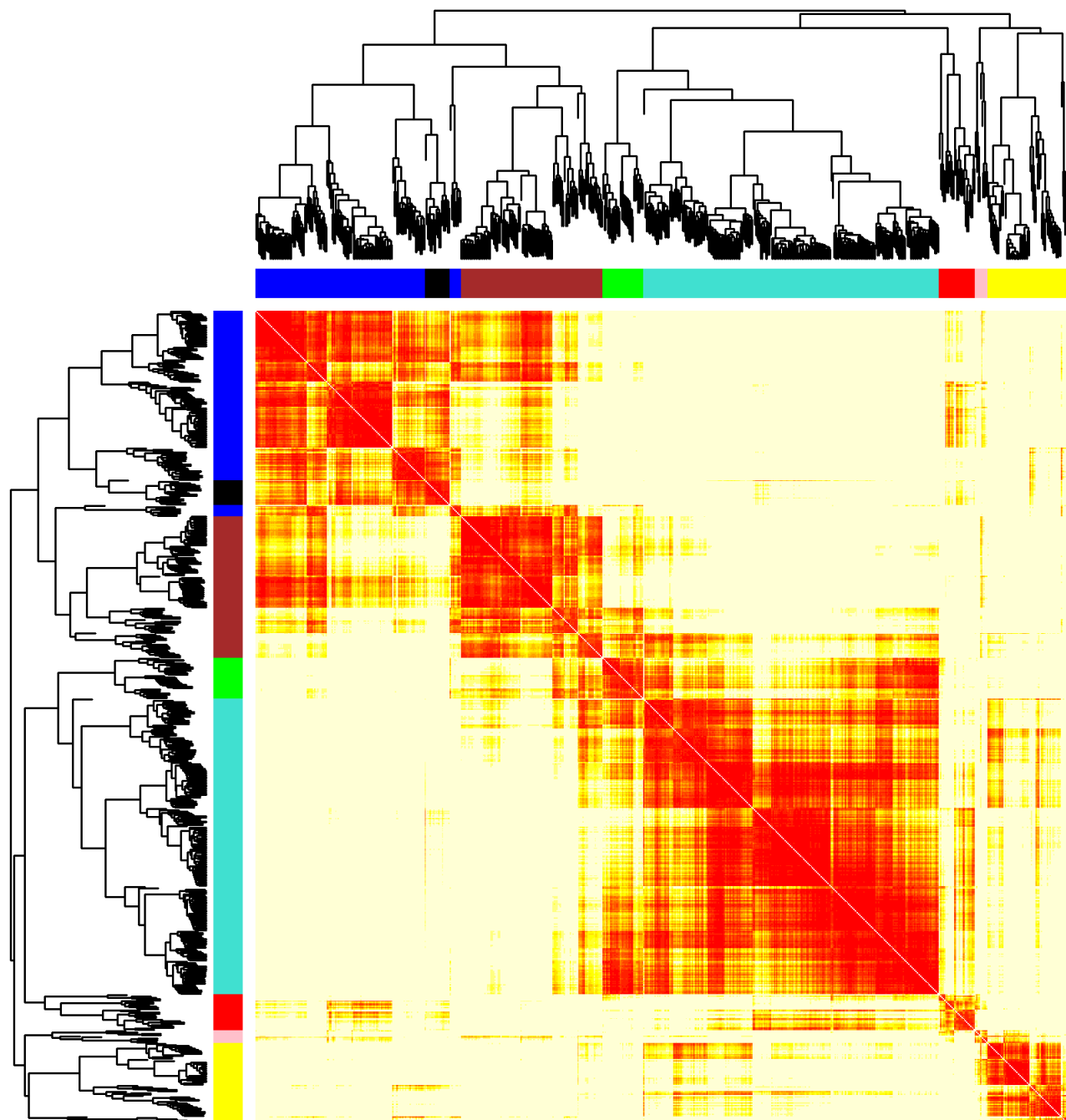

**Figure S6**

Supplement: Figure S6 — Specific modules identified associated with Dex or VD3 treatment by weighted gene co-expression network analysis (WGCNA). (PDF) [file pone.0060213.s006.pdf]

## A GO.BP annotation network

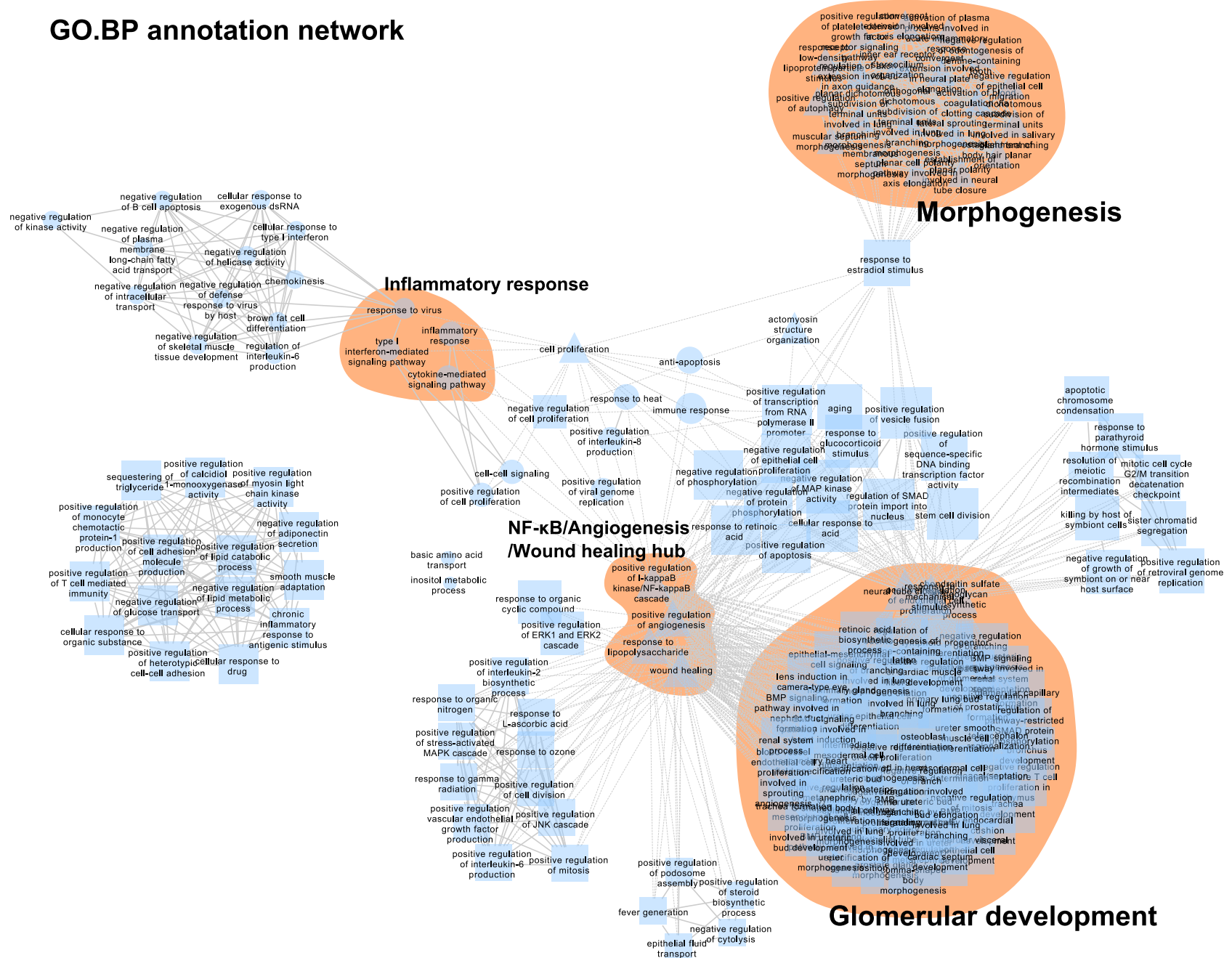

## B

### GO.CC annotation network

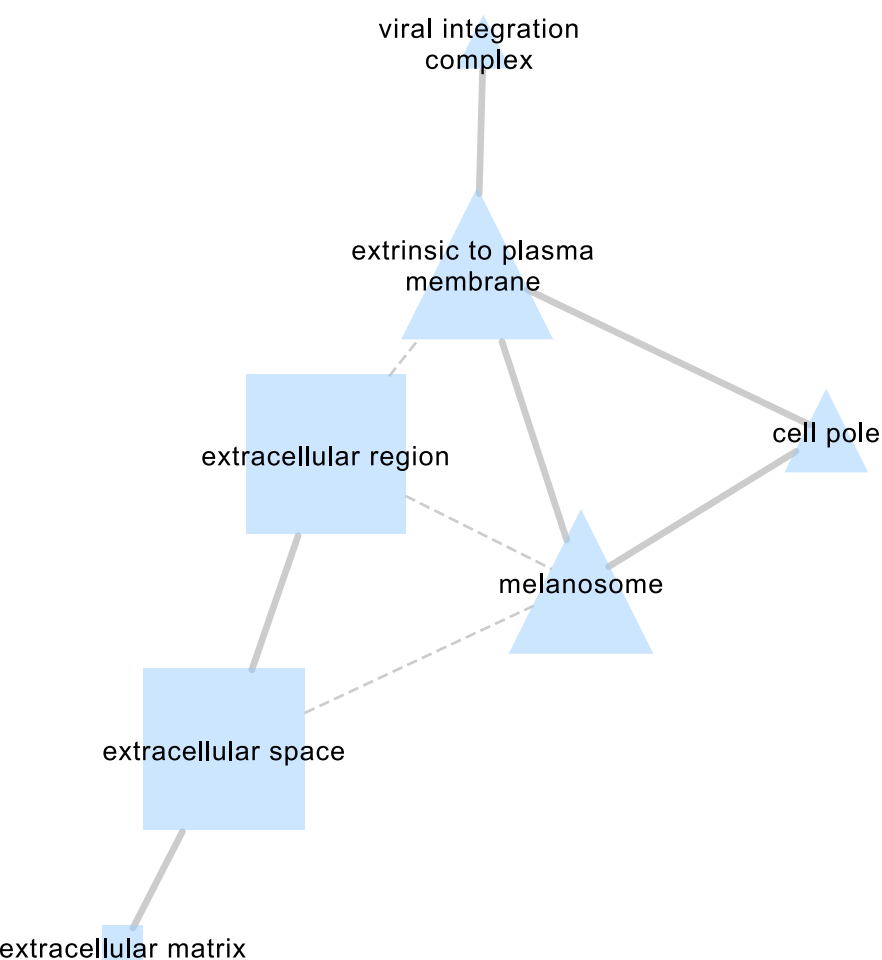

**C**

**KEGG annotation network**

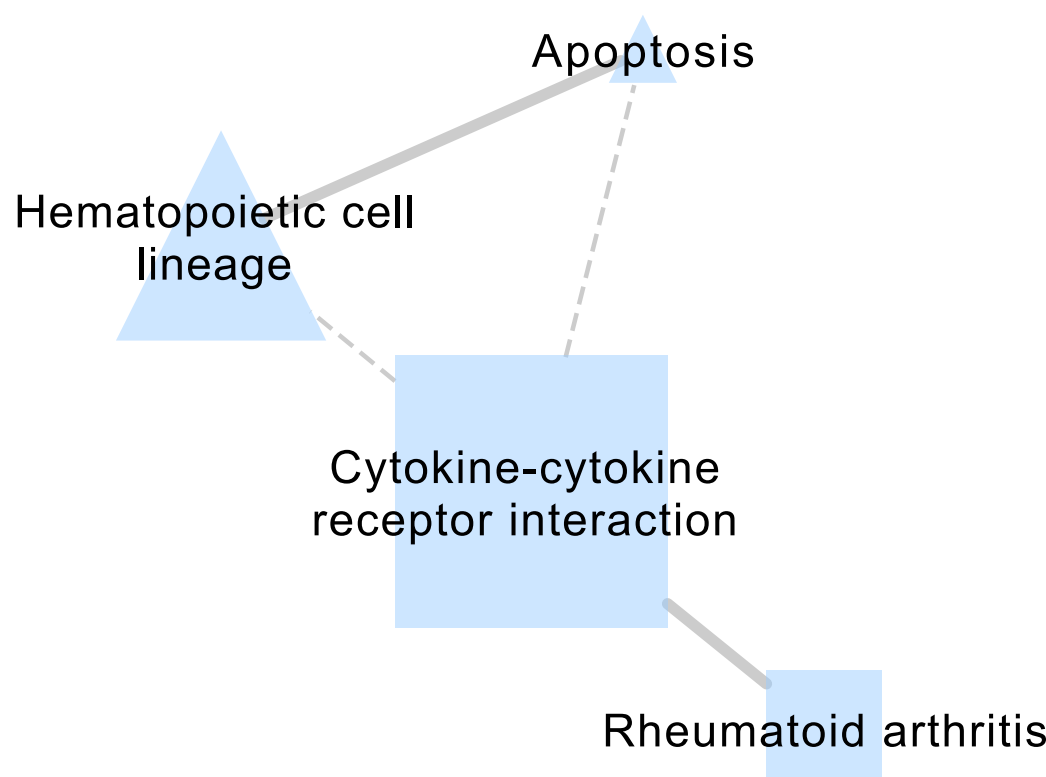

## Figure S7

Supplement: Figure S7 — Functional weighted networks of the significantly enriched annotations for the Dex treatment associated modules identified by WGCNA analysis. (PDF) [file pone.0060213.s007.pdf]

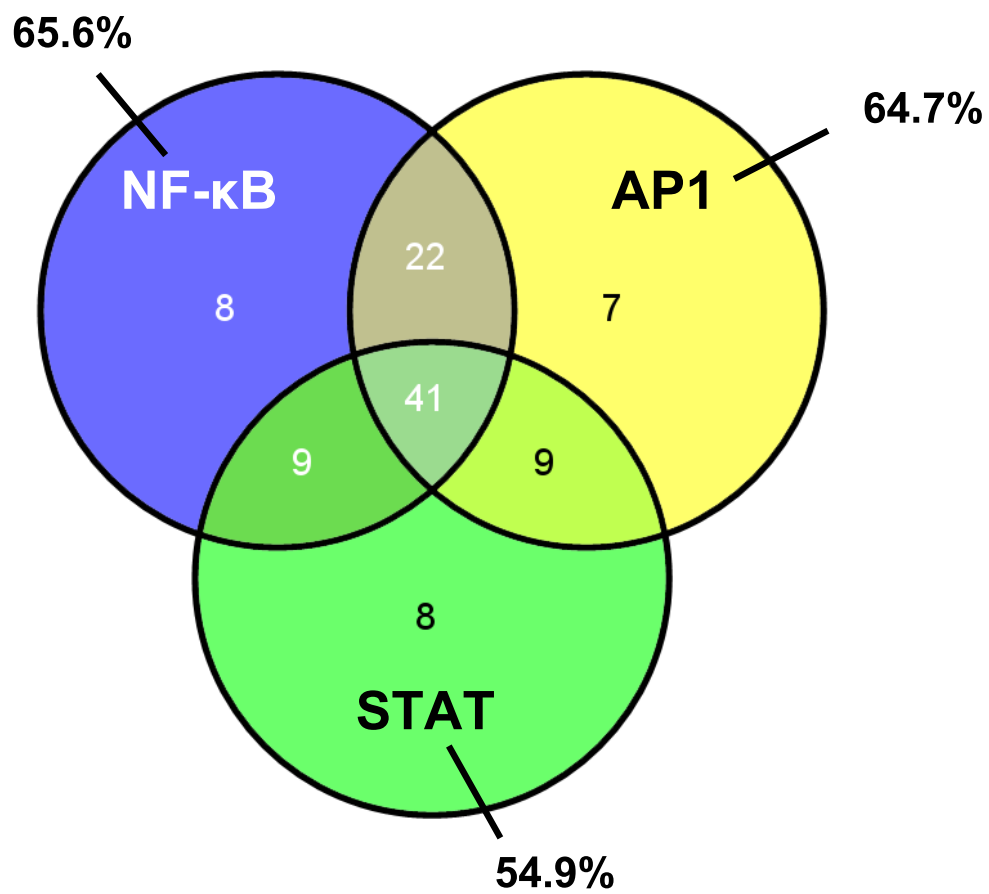

**Figure S8**

Supplement: Figure S8 — A profile of the significantly changed genes by Dex treatment during differentiation (D.Dex-D.Veh) regulated by known transcriptional factors. (PDF) [file pone.0060213.s008.pdf]

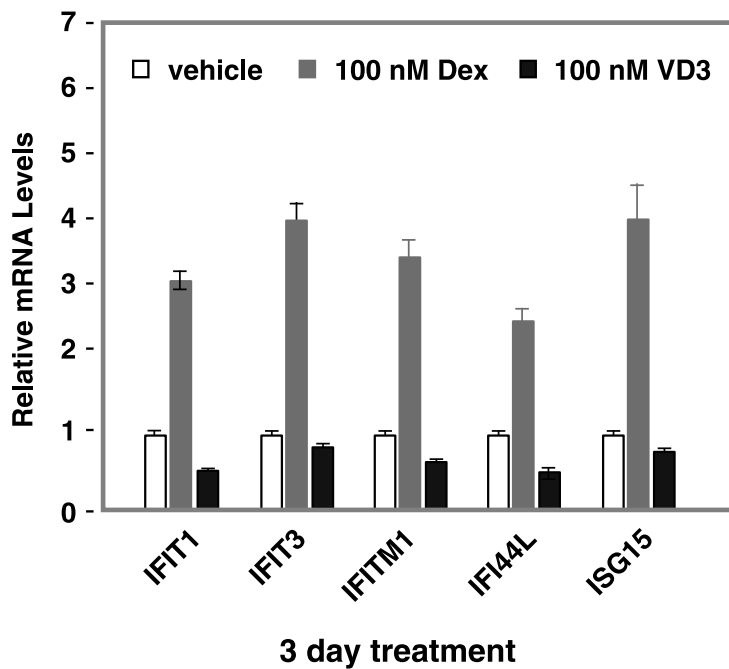

**Figure S9**

Supplement: Figure S9 — QRT-PCR validation of the mRNA accumulations of genes following Dex or VD3 treatment. (PDF) [file pone.0060213.s009.pdf]
