# Supplementary material for: Microarray Analyses of Glucocorticoid and Vitamin D3 Target Genes in Differentiating Cultured Human Podocytes
Source: PLoS One. 2013 Apr 4;8(4):e60213. doi: 10.1371/journal.pone.0060213 (PMC3617172; doi:10.1371/journal.pone.0060213)
Supplement: Table S7 — The sequences of primers used in this study. (DOCX) [file pone.0060213.s016.docx]

Supplementary Table 6:

| Gene name | Forward primers (5'-3') | Reverse primers (5'-3') |
| --- | --- | --- |
| *ANGPTL4* | TTGACCCGGCTCACAATGTC | TCCAGCCTCCATCTGAGGTC |
| *CCL2* | CTCGCTCAGCCAGATGCAAT | TTGGGTTTGCTTGTCCAGGT |
| *CCL20* | TGTCAGTGCTGCTACTCCAC | GTGTGAAAGATGATAGCATTGATGT |
| *DCN* | CCTTTGGTGAAGTTGGAACG | CGCAGCTCCTGAAGAGTTTT |
| *GAPDH* | GAAGGTGAAGGTCGGAGT | GAAGATGGTGATGGGATTTC |
| *GPR56* | CACCAGCTACAGCCGAAGAA | TGATGGTGTAGTCCCGAGGT |
| *GR-1** | GCTCTGGGGTGGAGATCATA | TGGTCGTACATGCAGGGTAG |
| *GR-2** | TGGAAGCTGTAAAGTTTTCTTCAA | TCTTCGAATTTTATCGATGATGC |
| *IFI44L* | GTTTTATGGCCACCGTCAGT | CTGGACTTTCCAGACCCAAC |
| *IFIT1* | AAAAGCCCACATTTGAGGTG | GAAATTCCTGAAACCGACCA |
| *IFIT3* | CAGTTGTGTCCACCCTTCCT | CTAGCAAAGCAGGCCATTTC |
| *IFITM1* | CTGATTCTGGGCATCCTCAT | TGTATCTAGGGGCAGGACCA |
| *IL1β* | AGCTACGAATCTCCGACCAC | CGTTATCCCATGTGTCGAAGAA |
| *ISG15* | TGTCGGTGTCAGAGCTGAAG | GCCCTTGTTATTCCTCACCA |
| *MEOX1* | AGGGCTACCCCACTACCCG | TCCTGGTTGTCTGAACTCTCCTT |
| *NRCAM* | CTCAAAATCTTGTGCTGTCCCC | GCAGTTCCCTGTTGTCCTTCAG |
| *SERPINE1* | CACCCTCAGCATGTTCATTG | AGCTGGGCACTCAGAATGTT |
| *SPP1* | GGCATCACCTGTGCCATACC | GACTTACTTGGAAGGGTCTGTG |
| *VDR-1** | GACTTTGACCGGAACGTGCCC | GACTTTGACCGGAACGTGCCC |
| *VDR-2** | CCAGTTCGTGTGAATGATGG | AGATTGGAGAAGCTGGACGA |

*Two different pairs of primers for GR or VDR were used for qRT-PCR validation.
