# Supplementary material for: Microarray Analyses of Glucocorticoid and Vitamin D3 Target Genes in Differentiating Cultured Human Podocytes
Source: PLoS One. 2013 Apr 4;8(4):e60213. doi: 10.1371/journal.pone.0060213 (PMC3617172; doi:10.1371/journal.pone.0060213)
Supplement: Methods S1 — All of the detailed methods and materials that are not covered by the main text are included in this supporting file. (DOCX) [file pone.0060213.s017.docx]

**Methods S1. Supporting methods and materials**

***Sample names***

The sample name abbreviation: U.Veh, undifferentiated with vehicle treatment; D.Veh, differentiated with vehicle treatment; D.VD3, differentiated with 100 nM VD3 treatment; D.Dex, differentiated with 100 nM Dex treatment.

***Multi-experiment viewer (MeV) analysis and expression terrain map***

MeV (v.4.8.1) is an open-source, R-based genomic data exploratory tool [1]. Raw scale data were imported and rescaled to row Z-score through the “Normalize Genes/Rows” function in the “Adjust Data” menu. The 3-D terrain map of sample relationship was generated using “Expression Terrain Map (TRN)” function under “Data Reduction” menu. The linkage line threshold was set at 0.9 and the redness of the line was positively scaled to the correlation coefficient.

***Venn diagram***

All venn diagrams were generated with “venny”, an online interactive tool which can be found at: http://bioinfogp.cnb.csic.es/tools/venny/index.html.

***DAVID analysis of functional annotation enrichment***

Database for Annotation, Visualization and Integrated Discovery (DAVID Bioinformatics Resources 6.7, <http://david.abcc.ncifcrf.gov/>) [2] was used to analyze the enrichment of the functional annotation information of a given gene list. To determine the converged annotation categories, “Functional Annotation Clustering” was performed with “Classification Stringency” set as “High”. The default functional annotation categories and the UCSC_TFBS (the ENCODE transcription factor binding site database through UCSC genome browser) were included in the analysis. The enrichment scores (-log10(*p* value)) of influenced categories were plotted as a barchart (Figure 1F) or as a heatmap (Figure 1D) based on a previous publication [3].

***Expression heatmap and subclusters identification***

The row (gene) dendrogram was calculated by Diana clustering algorithm while the column (sample) dendrogram by Hierarchical clustering algorithm in R [4,5]. The expression data was scaled by row (row Z-score) and plotted as a heatmap with green-dark-red scheme based on the sequences of the dendrograms. Sub-clusters were identified by the *dynamicTreeCut* R package [6] and annotated by the color side-bar.

***Weighted Gene Co-Expression Network Analysis (WGCNA)***

The analysis of weighed gene co-expression was performed using *WGCNA* R package [7] with soft-threshold power set at 20 that has been previously determined following the package instructions. The weighed gene co-expression modules were identified and annotated as color bars along the dendrogram. The interconnectivity of modules was visualized in the correlation coefficient based heatmap. The independent modules are represented as isolated box along the diagonal. The correlation coefficients between module and traits were calculated. The most interesting modules were identified as modules with high correlation coefficients and significant *p* values.

***Functional analysis of transcriptional networks***

The tested gene list was loaded to FUNNET (INSERM, <http://www.funnet.info/>) for network analysis of the top influenced annotation categories and related genes [8]. The annotation databases include Gene Ontology Biological Process (GO.BP), Gene Ontology Cellular Component (GO.CC), Gene Ontology Molecular Function (GO.MF) and Kyoto Encyclopedia of Genes and Genomes (KEGG). The network was visualized in Cytoscape (v.2.7.0), an open source network analysis and visualization software [9]. For the annotation network, the node size was scaled to centrality degree and the node shape reflects the membership of different gene co-expression network modules. For gene networks, the node centrality was measured by functional centrality and the scaled expression data was overlaid on the node with a green-red scheme. For more details, please refer to the softwares’ online documentation.

***Customized Entrez keyword analysis***

The customized keyword enrichment analysis was done with *GeneAnswers* R package [10]. Briefly, the Entrez-based gene network was retrieved using the customized keywords provided. The significantly changed gene list was tested against the Entrez keyword-based gene network through a Fisher’s exact test. The enrichment *p* values were reported. The tabular heatmap was generated to present the gene expression and Entrez keyword assignment.

**References**

1. Chu VT, Gottardo R, Raftery AE, Bumgarner RE, Yeung KY (2008) MeV+R: using MeV as a graphical user interface for Bioconductor applications in microarray analysis. Genome Biol 9: R118.

2. Huang da W, Sherman BT, Lempicki RA (2009) Systematic and integrative analysis of large gene lists using DAVID bioinformatics resources. Nat Protoc 4: 44-57.

3. Turnbaugh PJ, Ley RE, Hamady M, Fraser-Liggett CM, Knight R, et al. (2007) The human microbiome project. Nature 449: 804-810.

4. Gentleman RC, Carey VJ, Bates DM, Bolstad B, Dettling M, et al. (2004) Bioconductor: open software development for computational biology and bioinformatics. Genome Biol 5: R80.

5. RDC T (2009) R: A Language and Environment for Statistical Computing. Vienna, Austria: R Foundation for

Statistical Computing.

6. Langfelder P, Zhang B, Horvath S (2008) Defining clusters from a hierarchical cluster tree: the Dynamic Tree Cut package for R. Bioinformatics 24: 719-720.

7. Langfelder P, Horvath S (2008) WGCNA: an R package for weighted correlation network analysis. BMC Bioinformatics 9: 559.

8. Prifti E, Zucker JD, Clement K, Henegar C (2008) FunNet: an integrative tool for exploring transcriptional interactions. Bioinformatics 24: 2636-2638.

9. Smoot ME, Ono K, Ruscheinski J, Wang PL, Ideker T (2011) Cytoscape 2.8: new features for data integration and network visualization. Bioinformatics 27: 431-432.

10. Feng G, Du P, Krett NL, Tessel M, Rosen S, et al. (2010) A collection of bioconductor methods to visualize gene-list annotations. BMC Res Notes 3: 10.
